# Telemedicine in polish primary care during and after the COVID-19 crisis: a retrospective analysis of over 720,000 consultations

**DOI:** 10.3389/fpubh.2025.1695625

**Published:** 2025-11-21

**Authors:** Krzysztof Marcin Zakrzewski, Paulina Mularczyk-Tomczewska, Tytus Koweszko, Anna Mosiołek, Andrzej Silczuk

**Affiliations:** 1 The Independent Group of Public Ambulatory Care Institutions Warsaw-Ochota, Warsaw, Poland; 2 Department of Public Health, Faculty of Life Sciences, Medical University of Warsaw, Warsaw, Poland; 3 Department of Community Psychiatry, Faculty of Life Sciences, Medical University of Warsaw, Warsaw, Poland; 4 Department of Interdisciplinary Disability Studies, The Maria Grzegorzewska University of Special Education, Warsaw, Poland

**Keywords:** telemedicine, primary care, COVID-19, Poland, healthcare delivery, teleconsultations

## Abstract

**Background:**

The COVID-19 pandemic fundamentally reshaped healthcare delivery worldwide, accelerating the adoption of telemedicine. This study aimed to examine patterns of teleconsultation use in Polish primary care across pandemic and post-pandemic phases.

**Methods:**

We retrospectively analysed anonymized medical records from a nationwide primary care network (*N* = 54,430 patients; 720,133 consultations, January 2020–December 2024). The dataset comprised 507,668 in-person visits (70.5%) and 212,465 teleconsultations (29.5%). Variables included patient age, sex, consultation type, and clinical actions (prescriptions, referrals, diagnostic tests).

**Results:**

Teleconsultations accounted for 29.5% of all visits (*n* = 212,465). Before the pandemic, all consultations were in-person, whereas during lockdown teleconsultations peaked at 78.5% (*n* = 35,840). Their share declined to 65.1% in the first wave and 18.5% in the second wave, then stabilized at 11.8% (*n* = 18,223) during the state of epidemic threat and 14.9% (*n* = 32,153) in the post-COVID phase. Differences between periods were statistically significant (e.g., lockdown vs. pre-COVID: *χ*^2^ = 46,451.9, *p* < 0.001; post-COVID vs. pre-COVID: *χ*^2^ = 5,454.5, *p* < 0.001). Younger and middle-aged adults used teleconsultations proportionally more often than those ≥60 years, who consistently preferred in-person care. Remote visits were more frequently associated with prescription issuance, whereas in-person consultations more often involved diagnostic tests or specialist referrals.

**Conclusion:**

Teleconsultations surged to nearly 80% of visits during lockdown and stabilized at 12–15% post-pandemic. Persistent demographic disparities and modality-specific clinical profiles highlight the need for tailored strategies, strengthened digital infrastructure, and clear guidelines to ensure safe and equitable integration of telemedicine into routine primary care.

## Introduction

The global spread of the SARS-CoV-2 virus resulted in a dramatic increase in infections and deaths, leading to considerable disruptions in healthcare systems worldwide (1). According to the WHO pulse survey, access to essential health services declined by 5% to as much as 50%, with substantial variability between regions and healthcare systems. For example, some high-income European countries reported moderate declines in outpatient and elective care of around 5–15%, whereas many low- and middle-income countries experienced severe disruptions exceeding 40–50%, particularly in maternal and child health services (2). Vulnerable groups such as the older population, chronically ill individuals, and children were disproportionately affected. The World Health Organization (WHO) has warned that even minor disruptions in healthcare access may lead to increased morbidity and mortality from non-COVID-related causes (2). This concern is supported by evidence showing excess mortality linked to delays in cancer treatment (3) and worsening outcomes in chronic disease management (4), and alcohol use disorder (5).

At the same time, the pandemic accelerated the development and implementation of telemedicine across multiple areas of medical practice (6). In the context of restricted access to traditional services, remote healthcare became a key tool to ensure continuity of treatment (7). According to the WHO, telemedicine refers to the delivery of healthcare services at a distance by health professionals using information and communication technologies for diagnosis, treatment, prevention, and education (8). A defining feature of telemedicine is the separation between patient and provider, contributing to the improvement of individual and community health (9).

Although telemedicine predates the digital age, with early practices linked to the telegraph and radio, its broader recognition emerged in the second half of the 20th century. Initially applied in geographically isolated contexts such as rural (10–13), maritime (14–17), and space settings (18–20), telemedicine proved effective in overcoming barriers related to distance and limited infrastructure. Since the 1990s, advances in the Internet and digital technologies have expanded its capabilities to include remote diagnostics, monitoring, and automated procedures requiring minimal patient involvement (21).

In Poland, telemedicine remained marginal before 2020 due to insufficient legal frameworks, digital infrastructure, and staff competencies. A turning point came in 2020 with the COVID-19 pandemic, when new legal provisions enabled large-scale remote service delivery. Teleconsultations became mandatory in primary healthcare, and their number surged to 56.8 million in primary care and 16.3 million in specialist care in 2020; in some periods, they accounted for up to 80% of all patient encounters (22).

In the Polish legal framework, telemedicine is explicitly recognized as a form of medical activity under Article 3(1) of the Act on Medical Activity, which permits the provision of healthcare services via ICT systems (23, 24). During the pandemic, telemedicine was deployed on an unprecedented scale in primary healthcare (25). A legal definition of teleconsultation was subsequently introduced in §2(3) of the Regulation of the Minister of Health of 12 August 2020, defining it as a “healthcare service provided remotely using ICT systems or communication technologies” (26). With ongoing technological progress and the experience gained during the pandemic, further institutionalization of remote care is expected (24).

This study adds to existing evidence by providing a large scale, long term analysis of 720,133 primary care consultations in Poland, covering both pandemic and post pandemic phases. Specifically, we sought (a) to examine long term trends and demographic differences in the use of telemedicine versus in person visits, and (b) to investigate how consultation modality was associated with clinical decision making, including prescription issuance, referrals, and diagnostic testing, across distinct phases of the COVID-19 pandemic and the post pandemic period. By linking consultation modality with clinical decisions and demographic disparities, our analysis offers novel insights into the sustained integration of telemedicine and its implications for health policy and equitable access.

## Materials and methods

### Patient population

This retrospective study was based on anonymized medical records from all available patients of a primary healthcare network within the Independent Group of Public Ambulatory Care Institutions Warsaw Ochota, Poland, covering the period from January 2020 to December 2024. The network serves a large and demographically diverse urban and suburban population of approximately 160,000 to 170,000 residents. It includes several outpatient facilities that provide a broad range of primary care services, ensuring both geographic and service diversity within the Warsaw metropolitan area and its surroundings. Although the data come from a single administrative area, the patient population structure in terms of sex distribution and age range is broadly comparable to the demographic composition of primary care users in large Polish cities, as reported in national health statistics. However, no nationwide demographic registry of primary care users is available, and thus the generalizability of findings beyond this setting should be interpreted with caution. The dataset comprised 720,133 consultations provided to 54,430 individual patients. Patients ranged in age from 0 to 106 years (mean 55.6 years, SD = 27.7), and women constituted 59.4% of the study population (*n* = 427,456).

### Data collection

For each consultation, demographic information (age, sex), date of visit, type of service, and clinical outcomes (prescriptions issued, referrals to specialist care, diagnostic test orders) were collected. In total, the dataset included in-person visits (*n* = 507,668; 70.5%) and teleconsultations (*n* = 212,465; 29.5%). Data were aggregated according to distinct phases of the COVID-19 pandemic, as defined by national epidemic status declarations: pre-pandemic, epidemic threat state, lockdown, first wave, second wave, post-pandemic epidemic threat state, and post-COVID period.

This retrospective study was based on anonymized medical records from all available patients of a primary healthcare network within the Independent Group of Public Ambulatory Care Institutions Warsaw-Ochota.

Pandemic phases were defined based on official epidemic status declarations and major public health interventions implemented in Poland during the COVID-19 crisis.

### Variables

The main variable of interest was the consultation modality, categorized as either in-person or teleconsultation. Patient demographic characteristics (age group, sex) and consultation phase (according to epidemic status) were included as independent variables. Clinical outcomes recorded in the dataset included prescription issuance, referrals to specialist care, and diagnostic test orders.

### Statistical analysis

A retrospective observational design was applied to assess trends in the use of telemedicine during the COVID-19 pandemic and the subsequent post-pandemic period. Descriptive statistics were used to characterize the study population and the distribution of consultation types across pandemic phases. Associations between categorical variables (type of visit, pandemic phase, sex, age group) were evaluated using chi-square tests with Yates’ correction where appropriate, while the McNemar test assessed paired changes in patient preferences between phases. The dataset did not include patient-reported outcomes or qualitative indicators of individual preferences; the analysis was therefore limited to observed utilization patterns of consultation modalities. A retrospective observational design was applied to assess trends in the use of telemedicine during the COVID-19 pandemic and the subsequent post-pandemic period. Descriptive statistics were used to characterize the study population and the distribution of consultation types across pandemic phases. Associations between categorical variables (type of visit, pandemic phase, sex, age group) were evaluated using chi-square tests with Yates’ correction where appropriate, while the McNemar test assessed paired changes in patient preferences between phases. The dataset did not include patient-reported outcomes or qualitative indicators of individual preferences; the analysis was therefore limited to observed utilization patterns of consultation modalities.

In addition, logistic regression models were used to assess the influence of patient sex and visit type (teleconsultation vs. in-person) on clinical decisions, including prescription issuance, medical referrals, and diagnostic test orders. Separate models were constructed for each outcome across distinct pandemic phases. Separate models were constructed for each outcome across distinct pandemic phases. All results referred to individual consultations, regardless of patient identity or the number of visits recorded during the study period. Regression coefficients (B) and intercepts (B₀) were available for selected models and are reported where applicable. For models where these parameters were not accessible due to system-level export limitations, results are presented using odds ratios (OR), 95% confidence intervals (CI), χ^2^ statistics, and *p*-values. All models were based on complete cases; consultations with missing values for key variables (age, sex, visit type, clinical actions) were excluded from analysis. No imputation was performed.

Pandemic phases were defined according to official declarations by the Polish Ministry of Health and Chief Sanitary Inspectorate, which directly influenced healthcare service protocols. These phases included: pre-pandemic, epidemic threat (phase I), lockdown, first wave, second wave, epidemic threat (phase II), and post-COVID period. While administrative in origin, these phases reflect real-world shifts in service delivery and patient behavior, and were therefore used as stratification variables in the analysis.

All statistical analyses were conducted using Statistica 13.3 (StatSoft Inc., Tulsa, OK, USA) under the institutional license of the Medical University of Warsaw.

All models were based on complete cases; consultations with missing values for key variables (age, sex, visit type, clinical actions) were excluded from analysis. No imputation was performed. Basic data validation was applied to ensure internal consistency and remove implausible or duplicate entries. These steps preserved the integrity of the dataset while maintaining its real-world structure.

All statistical analyses were conducted using Statistica 13.3 (StatSoft Inc., Tulsa, OK, USA) under the institutional license of the Medical University of Warsaw.

### Ethical considerations

All patient identifiers were removed prior to analysis. The study protocol complied with ethical and legal standards for the use of retrospective healthcare data and was conducted in accordance with the ethical principles outlined in the Declaration of Helsinki. The research protocol received approval from the Ethics Committee of the Medical University of Warsaw (decision no. AKBE/188/2025, issued on 30 June 2025).

## Results

A total of 720,133 primary care consultations were analyzed for 54,430 patients between 2020 and 2024. Table 1 presents the demographic characteristics of the study population.

**Table 1:** Characteristics of the study group (*N* = 720,133).

	Min–Max (years)	Average	Departure Std.
Age	0–106	55,634	27,691
		*N*	%
Sex	Woman	427,456	59,366
	Man	292,677	40,634

*N*, number of subjects.

### Analysis of the frequency of teleconsultations and in-person visits

**Figure 1:**
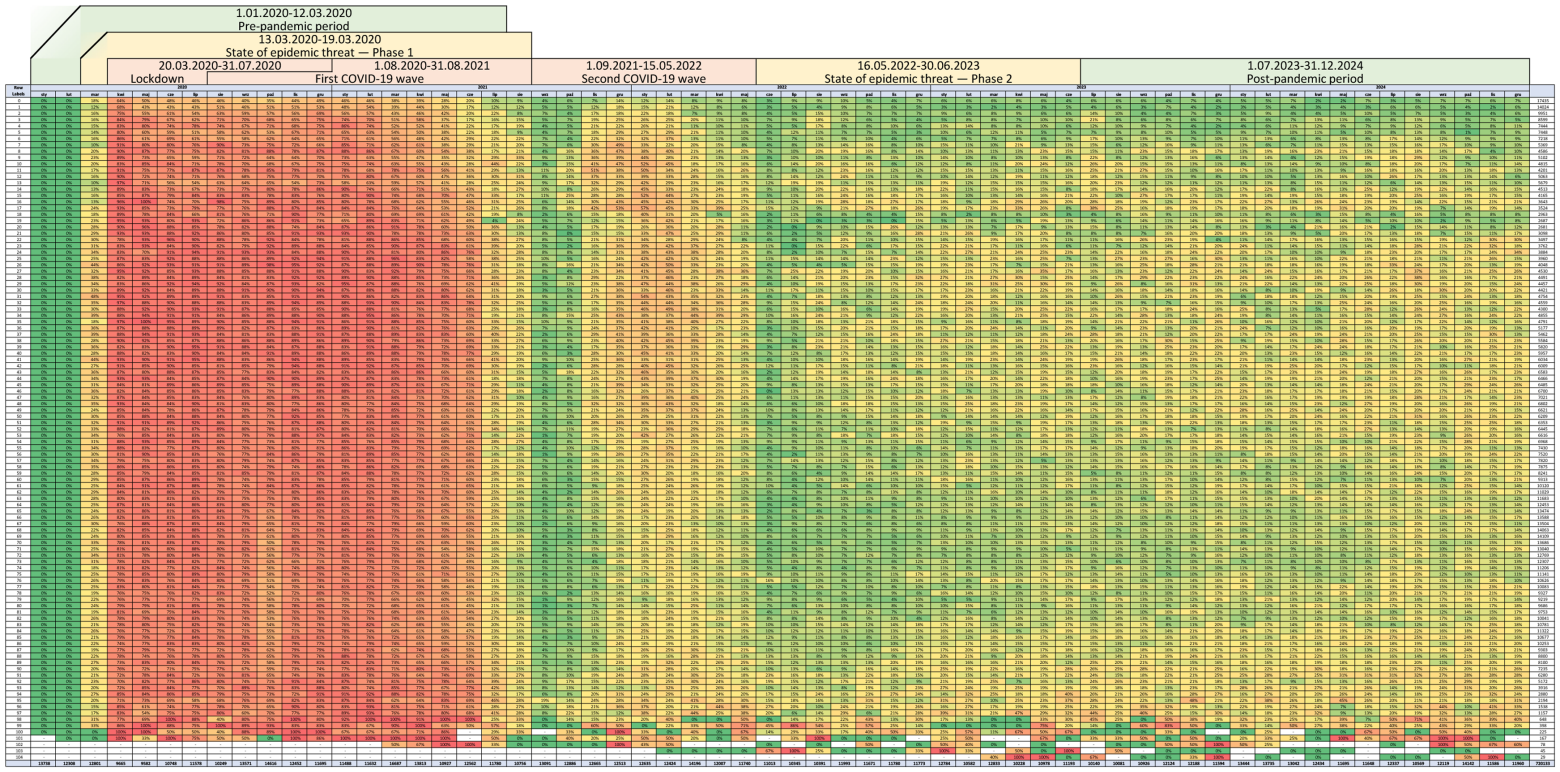
Distribution of teleconsultations and in-person visits across phases of the COVID-19 pandemic in the total study population (number and percentage).

Teleconsultations, virtually absent before 2020, constituted 78.4% of all visits during the lockdown period and remained part of service provision throughout subsequent phases. In the post-pandemic period, their share ranged from 11.8 to 14.9% of all primary care encounters. The distribution of teleconsultations and in-person visits across distinct phases of the COVID-19 pandemic within the total study population is presented in Table 2 and Figure 1.

**Table 2:** Distribution of teleconsultations and in-person visits across phases of the COVID-19 pandemic in the total study population (number and percetage).

Pandemic COVID-19 phases	Teleconsultations		In-person visits	
	*N*	%	*N*	%
Generally	212,465	29.5	507,668	70.5
Pre-COVID	0	0	31,850	100
Epidemic threat state I	786	27.3	2091	72.4
Lockdown	35,840	78.5	9,853	21.5
First wave	105,556	65.1	56,672	34.9
Second wave	19,907	18.5	87,488	81.5
Epidemic threat state II	18,223	11.8	136,102	88.2
Post-COVID	32,153	14.9	183,612	85.1

**Figure 2:**
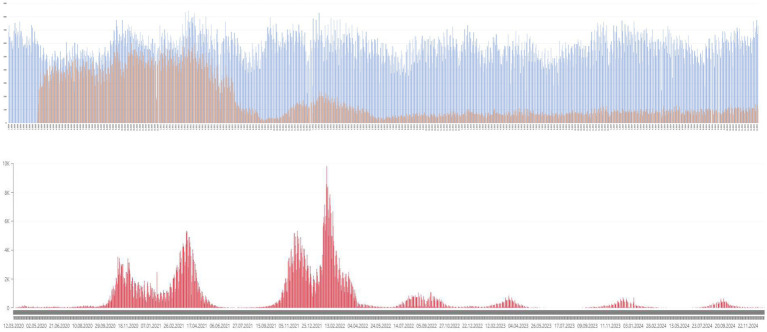
Graphical representation of the proportion of telemedicine visits (oragne) among all outpatient consultations (blue) in relation to the incidence of new COVID-19 infections (red) during the first and second waves.

The number and proportion of teleconsultations and in-person visits across age groups and epidemic phases, including the lockdown, first and second waves, post-threat state, and post-COVID periods, are presented in Table 3 and Figure 2. The pre-pandemic phase was excluded from this stratification, as all visits during that time were conducted in person.

**Table 3:** Age-stratified distribution of teleconsultations and in-person visits across COVID-19 epidemic phases in the total study population (*N* and %).

Age range (years)	Visit type	Threat state (pre)		Lockdown		First wave		Second wave		Threat state (post)		Post-COVID	
		*N*	%	*N*	%	*N*	%	*N*	%	*N*	%	*N*	%
0–17	TC IPV	44 239	15.5 84.5	4,019 2,326	63.3 36.7	13,906 13,944	49.9 50.1	3,523 15,816	18.2 81.8	2,607 23,488	10.0 90.0	3,716 31,850	10.4 89.6
18–30	TC IPV	75 128	36.9 63.1	2,378 339	87.5 12.5	8,380 2,683	75.7 24.3	1982 5,942	25.0 75.0	1,477 8,526	14.8 85.2	2,397 11,904	16.8 83.2
31–45	TC IPV	121 236	33.9 66.1	4,066 569	87.7 12.3	14,866 4,594	76.4 23.6	3,576 9,455	27.4 72.6	2,703 14,900	15.4 84.6	4,435 20,207	18.0 82.0
46–60	TC IPV	163 380	30.0 70.0	5,654 1,152	83.1 16.9	17,055 7,156	70.4 29.5	3,231 12,351	20.7 79.3	2,830 20,473	12.1 87.9	5,378 27,851	16.2 83.8
60+	TC IPV	383 1,108	25.7 74.3	19,723 5,467	78.3 21.7	51,349 28,295	64.5 35.5	7,595 43,924	14.7 85.3	8,606 68,715	11.1 88.9	16,227 91,800	15.0 85.0

TM, telemedicine; IP-V, in person visit; *N*, number.

### Pre-COVID and pre-pandemic epidemic threat state

During the epidemic threat state, 27.32% of patients used teleconsultations, while 72.68% chose in-person visits. In the pre-COVID period, all visits were conducted in person. Chi-square analysis showed a significant difference between the two periods (*χ*^2^ = 8471.85, *p* < 0.001; *Φ* = 0.244). The Yates-corrected chi-square test (*χ*^2^ = 8459.32, p < 0.001; Φ = 0.244) and McNemar test (*χ*^2^ A/D = 2089.00, *p* < 0.001) confirmed significant differences in the distribution of visit types between these periods.

### Pre-COVID and lockdown

During the lockdown, 78.5% of all visits were teleconsultations, compared to 100% in-person visits before the pandemic. Chi-square testing indicated significant differences between the pre-pandemic and lockdown periods (*χ*^2^ = 46451.9, *p* < 0.001; *χ*^2^ Yates = 46448.7, *p* < 0.001). The McNemar test also showed a significant change in visit utilization patterns (*χ*^2^ A/D = 9851.00, *p* < 0.001). The Phi coefficient suggested a strong association between pandemic period and type of visit (*Φ* = 0.599).

### Pre-COVID and the first wave of the pandemic

During the first wave, 65.07% of all visits were teleconsultations. In the pre-COVID period, all visits were in-person. Statistical analyses demonstrated significant differences (*χ*^2^ = 45435.1, *p* < 0.001; *χ*^2^ Yates = 45432.5, *p* < 0.001). The Phi coefficient indicated a moderate association (Φ = 0.234). McNemar testing confirmed differences in utilization patterns (*χ*^2^ A/D = 56670.0, *p* < 0.001).

### Pre-COVID and the second wave of the pandemic

During the second wave, in-person visits accounted for 81.46% of all visits, while 18.54% were teleconsultations. Compared to the pre-pandemic period (100% in-person), the difference was statistically significant (*χ*^2^ = 6888.62, *p* < 0.001; *χ*^2^ Yates = 6887.10, *p* < 0.001). The Phi coefficient indicated a weak association (*Φ* = 0.049). The McNemar test confirmed a significant change in distribution (*χ*^2^ A/D = 87486.0, *p* < 0.001).

### Pre-COVID and post-pandemic epidemic threat state

During the post-pandemic epidemic threat state, in-person visits accounted for 88.19% of all consultations and teleconsultations for 11.81%. Compared to the pre-COVID period, the difference was statistically significant (*χ*^2^ = 4168.97, *p* < 0.001; *χ*^2^ Yates = 4167.64, *p* < 0.001). The Phi coefficient indicated a weak association (*Φ* = 0.022). The McNemar test confirmed significant differences (*χ*^2^ A/D = 136100.0, *p* < 0.001).

### Pre-COVID and post-COVID

In the post-COVID period, 85.10% of visits were in-person and 14.90% were teleconsultations. Compared to the pre-pandemic period, this difference was statistically significant (*χ*^2^ = 5454.52, *p* < 0.001; *χ*^2^ Yates = 5453.20, *p* < 0.001). The Phi coefficient indicated a weak association (*Φ* = 0.022). McNemar testing confirmed significant differences (*χ*^2^ A/D = 183610.0, *p* < 0.001).

### Logistic regression: sex as a determinant of visit type

Analysis of sex differences in visit mode across pandemic phases revealed consistent but generally small effects. During lockdown male patients had marginally higher odds of attending in-person visits than female patients (OR = 1.05, 95% CI 1.01–1.10; *χ*^2^ = 4.62, *p* = 0.032), with teleconsultations comprising 78.4% of encounters. In the first wave the association remained significant and slightly stronger (OR = 1.08, 95% CI 1.03–1.13; *χ*^2^ = 52.16, *p* < 0.001), while teleconsultations still represented the majority (65.1%). The second wave reflected a return to facetoface care (81.5% inperson), with men more likely to attend in person (OR = 1.15, 95% CI 1.11–1.19; *χ*^2^ = 75.93, *p* < 0.001). In the postpandemic threat period teleconsultations again predominated (88.2%) and men were less likely to use them (OR = 0.82, 95% CI 0.80–0.85; *χ^2^* = 146.81, *p* < 0.001). After the pandemic, in-person visits predominated (85.1%) and were more frequently utilized by men (OR = 1.18, 95% CI 1.16–1.21; *χ*^2^ = 185.04, *p* < 0.001).

### Prescription issuance

Across the entire period analyzed, sex and visit type had a statistically significant, albeit weak association with prescription issuance (*χ*^2^ = 702.73, *p* < 0.001). Men were less likely to receive prescriptions compared to women (OR = 0.88, 95% CI: 0.87–0.89), though the difference was relatively minor. The type of visit, whether teleconsultation or in-person did not significantly affect prescription issuance (OR = 0.97, 95% CI: 0.97–0.97), indicating that the consultation format was not a critical factor in prescribing decisions. During the lockdown phase, sex showed a significant effect, with men being less likely to receive prescriptions than women (OR = 0.86, 95% CI: 0.83–0.90). However, the type of visit had no meaningful association (OR = 0.999, 95% CI: 0.998–1.000). In the first wave, both sex and visit type were significant (*χ*^2^ = 186.72, *p* < 0.0001), with men less likely to receive prescriptions (OR = 0.87, 95% CI: 0.85–0.89) and in-person visits slightly increasing prescription likelihood (OR = 1.04, 95% CI: 1.02–1.06).

### Medical referrals

Analysis of medical referrals revealed that both sex and visit type had a statistically significant but weak association during the entire study period (*χ*^2^ = 53.96, *p* < 0.001). There was no significant sex difference (OR = 0.99, 95% CI: 0.99–0.99), and visit type had a minor relationship (OR = 0.96, 95% CI: 0.96–0.97). During the lockdown, the visit type significantly influenced referral issuance (*χ*^2^ = 17.24, *p* < 0.0002), with in-person visits more likely to result in referrals (OR = 0.91, 95% CI: 0.87–0.95). This trend continued during the first wave, with visit type being a significant factor (*χ*^2^ = 61.40, *p* < 0.0001), and in-person consultations leading to more referrals (OR = 0.92, 95% CI: 0.90–0.94).

### Diagnostic test orders

For diagnostic test orders, both sex and visit type had a statistically significant but very weak association throughout the entire period (*χ*^2^ = 35.99, *p* < 0.001). Sex had virtually no relationship on test ordering (OR = 0.998), and visit type also showed minimal influence (OR = 0.965). During the lockdown, both factors were significant (*χ*^2^ = 36.08, *p* < 0.0001), with men slightly more likely to receive test orders (OR = 1.03, 95% CI: 0.99–1.09), and in-person visits increasing the likelihood of test orders (OR = 0.85, 95% CI: 0.81–0.90). This pattern persisted in the first wave, with both sex and visit type showing only a negligible statistical association with test orders (*χ*^2^ = 55.28, *p* < 0.0001), where men were only marginally more likely to receive test commissions (OR = 1.01, 95% CI: 1.01–1.02).

## Discussion

In this study, we found that teleconsultations became a stable component of primary care after the pandemic, persisting at 11.8–14.9% of visits, with younger adults using them disproportionately more often, while older adults continued to rely on in-person consultations. The modality-specific clinical patterns were observed, with prescriptions more frequently issued during remote visits and diagnostic procedures initiated predominantly in face-to-face consultations. The sustained share of teleconsultations in Polish primary care following the pandemic indicates a structural rather than transient adaptation of healthcare delivery. Remote visits constituted 78.4% of all encounters during lockdown, and although their frequency decreased once restrictions were lifted, they stabilized at 11.8–14.9% in subsequent periods, reflecting a persistent role of telemedicine in service provision. The observed pattern mirrors broader international trajectories, yet the present study extends existing evidence by documenting continuity over nearly 5 years and linking consultation modality with demographic characteristics and clinical decision-making. Whereas younger and middle-aged adults were more likely to use teleconsultations, older individuals predominantly relied on in-person consultations, consistent with previous findings that digital barriers, rather than clinical unsuitability remain a key source of exclusion among older patients.

These findings are generally consistent with previous research. Studies from Israel have shown that, when adequately supported, older adults can sustain high levels of tele-health use beyond the acute crisis period (27), suggesting that low utilization among seniors may be more attributable to modifiable structural constraints than inherent preferences. This interpretation is reinforced by research indicating that digital literacy, infrastructure access, and targeted training programs significantly enhance the ability of older adults to benefit from remote care (28, 29).

Patterns of clinical decision-making further illuminate how telemedicine functioned in practice. Teleconsultations were more commonly associated with prescription issuance and renewal, whereas in-person visits more frequently resulted in diagnostic referrals or test orders. These findings are consistent with prior work demonstrating that orders initiated during remote visits are significantly less likely to be completed than those initiated in-person (adjusted OR ≈ 0.55) (30). The trend is similarly reflected in U. S.-based analyses showing lower rates of prescriptions, diagnostic testing, and imaging during tele-health encounters, as well as a greater likelihood of subsequent in-person follow-up (31). Taken together, this pattern underscores two interrelated constraints of remote care: the absence of physical examination and the limited immediate access to diagnostic pathways. At the same time, telemedicine does not appear inherently inferior in diagnostic performance; under clinically appropriate conditions, diagnostic concordance between remote and face-to-face visits in primary care settings can exceed 85% (32). Rather than suggesting a deficit of telemedicine per se, these results highlight the importance of clarifying which clinical contexts lend themselves to remote care delivery and which require tactile or proximity-based assessment.

The decline in teleconsultation use following the formal lifting of pandemic restrictions likely reflects not only behavioral readjustment but also institutional incentives. In mid-2021, amendments to NFZ contracting rules introduced higher reimbursement levels for in-person care relative to remote consultations (33, 34), which may have disincentivised providers from sustaining high telemedicine volumes. This policy shift coincided temporally with the observed decline and likely contributed to narrowing the role of teleconsultations to selected indications and patient subgroups.

However, observed utilization patterns cannot be interpreted solely through supply-side mechanisms. A range of unmeasured confounders including socioeconomic status, comorbidity, digital access, and educational attainment may have simultaneously shaped both modality choice and downstream clinical outcomes (35). Studies across healthcare systems have consistently shown that technological access, digital skills, and socioeconomic gradients remain persistent determinants of tele-health adoption (36–38). Furthermore, repeatedly documented barriers such as inadequate connectivity or lack of user competence disproportionately affect socially disadvantaged groups (39). These structural determinants imply that the observed demographic variation in teleconsultation uptake may reflect systemic inequality in digital enablement rather than patient preference alone.

The broader health systems perspective reinforces this interpretation. Sustaining telemedicine requires more than technological availability: it depends on equitable access and institutional embedding. As noted in prior analyses, without policies that explicitly bridge digital divides, tele-health risks exacerbating rather than alleviating existing health inequalities (40–42). The persistence of teleconsultations after the acute pandemic phase can be seen as a marker of adaptive capacity within the system, consistent with the conceptualization of resilience in health services (43). Yet the uneven uptake across age groups highlights that resilience is not uniformly distributed; where digital preparedness is lacking, tele-health operates as a selective rather than universal good (44). This underscores the importance of policy interventions that target digital literacy, technology infrastructure, and inclusive service design (45). The stabilization of teleconsultation rates at approximately 12–15% of all encounters suggests not merely residual pandemic behavior, but a reconfiguration of access pathways and clinical practice norms into a hybrid care model (46).

Interpreted from this perspective, telemedicine emerges not as a temporary substitute for conventional care, but as a durable component of a multi-modal primary care system one whose equity and reach will continue to depend on how effectively structural barriers to digital participation are addressed, as well as on sustained patient satisfaction and perceived quality of care, which have been shown to play a pivotal role in long-term telehealth uptake (47).

### Limitations of the study

This study has several limitations that should be considered when interpreting the results. First, the analysis was based on data from a single primary healthcare network in Poland, which may limit the generalizability of findings to other healthcare settings or regions with different organisational models. Second, the retrospective design and reliance on routinely collected medical records restricted the availability of potentially relevant variables, such as socioeconomic status, comorbidities, or patient-reported outcomes. Third, the classification of pandemic phases was based on official national epidemic status declarations, which may not fully capture the dynamic epidemiological context or regional variations in restrictions. Additionally, the study did not assess the quality of care, clinical effectiveness, or patient satisfaction in relation to teleconsultations versus in-person visits, which could provide further insights into the appropriateness of each modality. Finally, unmeasured confounding factors, including physician preference, local resource availability, and evolving telemedicine policies, may have influenced the observed trends.

This study was intentionally designed as a large-scale quantitative analysis focusing on utilization patterns, demographic differences, and clinical decision-making in primary care telemedicine. The use of data from a single primary care network may limit the generalizability of findings to other regions or healthcare systems. Indicators of care quality, such as clinical effectiveness, patient satisfaction, and guideline adherence, were not assessed, as the retrospective and administrative nature of the dataset did not permit their inclusion.

As such, aspects related to the quality and safety of care, as well as patient and provider satisfaction, were not assessed due to the retrospective nature of the dataset, which did not include patient-reported or qualitative indicators. In addition, full regression parameters (B, B₀) were available only for selected models and could not be consistently reported due to system-level export constraints.

### Practical implications

Our findings provide several important insights for the practical integration of telemedicine into primary care in Poland. First, the persistence of teleconsultations at a stable level of 11.8–14.9% of all primary care encounters in the post-pandemic phase indicates that remote consultations have become a permanent, though supplementary, component of healthcare delivery. This highlights the need to maintain appropriate infrastructure and clear organizational standards to support the safe and effective long-term use of telemedicine. Second, age-stratified results show that older adults consistently preferred in-person visits, whereas younger and middle-aged patients used teleconsultations more frequently. This disparity underscores the importance of developing targeted interventions to enhance digital accessibility and literacy among older populations, as well as ensuring alternative access pathways for those unable or unwilling to engage in remote care. Third, our data revealed that teleconsultations were more frequently associated with prescription issuance, while in-person visits more often led to diagnostic testing and specialist referrals. This pattern has implications for clinical workflows: remote care may be particularly suited to prescription renewals and follow-up of stable chronic conditions, whereas initial diagnoses and cases requiring physical examination remain more appropriate for in-person encounters. Recognizing these modality-specific clinical profiles can inform the development of guidelines that optimize the allocation of consultations according to patient needs and clinical complexity. Taken together, these findings suggest that telemedicine should be embedded as a complementary tool rather than a replacement for in-person visits. Policies that support equitable access, digital literacy programs, and evidence-based clinical standards will be essential to ensure that the observed changes in healthcare delivery translate into safe, effective, and inclusive long-term practice.

## Conclusion

The COVID-19 pandemic transformed primary care delivery in Poland, bringing telemedicine from a marginal to a mainstream modality. Although its use declined after the peak crisis periods, teleconsultations have remained a stable component of care, indicating structural adaptation rather than a temporary response. Utilization differed across age groups and clinical contexts, with remote visits predominating for prescription-related care and in-person consultations for diagnostics and referrals. These findings suggest that telemedicine should function as a complementary element of primary care within hybrid models. Ensuring equitable access will require continued investment in digital infrastructure and literacy, particularly for older adults. Appropriate reimbursement policies, professional training, and clear clinical guidelines will be essential to support safe and effective long-term integration of tele-health services.

## Data Availability

The raw data supporting the conclusions of this article will be made available by the authors, without undue reservation.
